# Pterocarpadiols A–D, Rare 6a,11b-Dihydroxypterocarpans from *Derris robusta*

**DOI:** 10.1007/s13659-015-0078-y

**Published:** 2015-11-07

**Authors:** Xiang-Mei Li, Mei-Fen Mao, Fu-Cai Ren, Xian-Jun Jiang, Ping Hai, Fei Wang

**Affiliations:** BioBioPha Co., Ltd., Kunming, 650201 People’s Republic of China

**Keywords:** *Derris robusta*, 6a,11b-Dihydroxypterocarpan, Pterocarpadiol

## Abstract

**Abstract:**

Four hitherto unknown 6a,11b-dihydroxypterocarpans, namely pterocarpadiols A–D (**1**–**4**), were isolated from the ethanol extract of the twigs and leaves of *Derris robusta*. Their structures were elucidated on the basis of extensive spectroscopic analysis. Pterocarpadiols A–D are a kind of very rare 6a,11b-dihydroxypterocarpans, and their presence as markers may be helpful in chemotaxonomical classification.

**Graphical Abstract:**

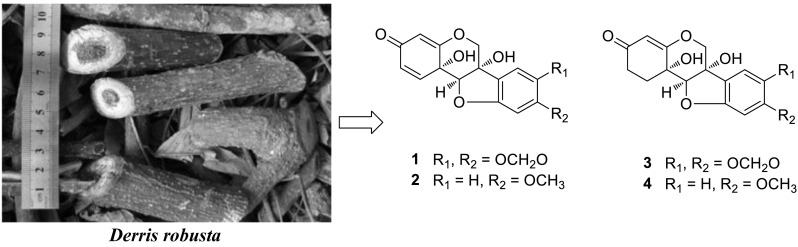

**Electronic supplementary material:**

The online version of this article (doi:10.1007/s13659-015-0078-y) contains supplementary material, which is available to authorized users.

## Introduction

The genus *Derris* (Leguminosae) contains about 800 species, widely distributed in tropical and subtropical parts of the world [1]. The bark and leaves of *Derris* species are commonly utilized as folk medicine to treat human diseases such as arthritis and eczema [2]. The extracts and reported chemical constituents exhibit antioxidant, antibacterial and pesticidal activities [2]. Previous studies show the genus is a rich source of flavonoids, isoflavonoids, pterocarpans and rotenoids [2]. As part of a BioBioPha [http://www.chemlib.cn] objective to assemble a large-scale natural product library very valuable in the discovery of new drug leads from nature [3–7], the phytochemical investigation on the twigs and leaves of *Derris robusta* led to the isolation of four new 6a,11b-dihydroxypterocarpans, namely pterocarpadiols A–D (**1**–**4**), together with 10 known pterocarpans: 1,11b-dihydro-11b-hydroxymaackiain (**5**) [8], 1,11b-dihydro-11b-hydroxymedicarpin (**6**) [8], pisatin (**7**) [9], variabilin (**8**) [10], 6a-hydroxymaackiain (**9**) [11], 6a-hydroxymedicarpin (**10**) [12], maackiain (**11**) [13], medicarpin (**12**) [14], 3-hydroxy-8,9-methylenedioxypterocarpene (**13**) [15], and anhydroglycinol (**14**) [16]. Among the known pterocarpans, compounds **5**–**10** and **14** were isolated from the genus for the first time. This paper reports the isolation and structure elucidation of pterocarpadiols A–D.

## Results and Discussion

**Fig. 1:**
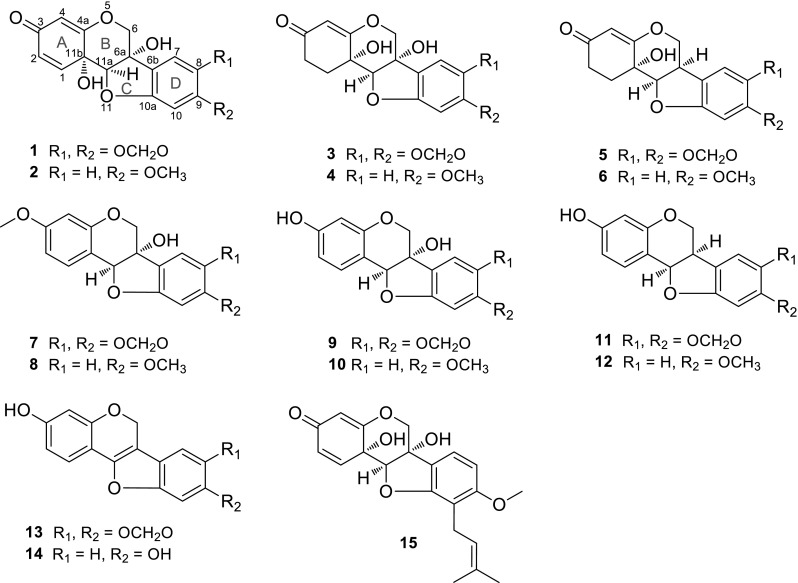
Pterocarpans from *Derris robusta* (**1**–**14**) and hydroxycristacarpone (**15**)

**Fig. 2:**
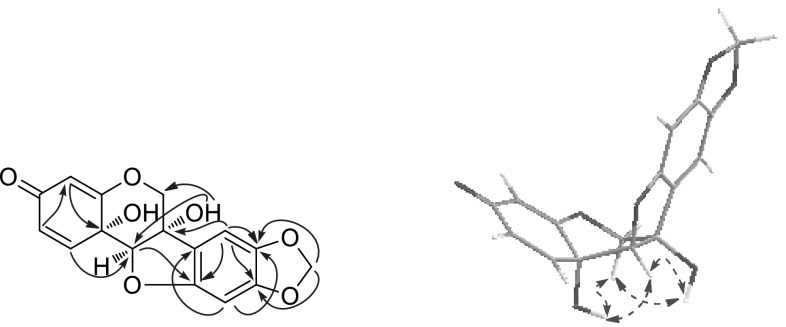
Key HMBC () and ROESY () correlations of pterocarpadiol A (**1**)

Compound **1**, obtained as amorphous powder, had a molecular formula of C_16_H_12_O_7_ as deduced from its positive-ion HRESIMS at *m/z* 339.0462 [M+Na]^+^ (calcd for C_16_H_12_O_7_Na, 339.0475), requiring 11 degrees of unsaturation. The ^1^H NMR spectrum (Table 1) showed three characteristic aliphatic protons of 6a-hydroxypterocarpan skeleton at *δ*_H_ 4.99, 4.37 (each 1H, d, *J* = 10.5 Hz, H-6), and 4.73 (s, H-11a) [17]. The ^13^C NMR spectrum (Table 3) displayed a total of 16 carbon resonances, including three typical oxygen-bearing carbons of 6a-hydroxypterocarpan at *δ*_c_ 70.2 (t, C-6), 78.7 (s, C-6a) and 91.4 (d, C-11a). Three olefinic protons at *δ*_H_ 6.80 (d, *J* = 10.0 Hz, H-1), 6.09 (dd, *J* = 10.0, 1.8 Hz, H-2), and 5.41 (d, *J* = 1.8 Hz, H-4) were assignable to A-ring by comparison with hydroxycristacarpone (**15**) (Fig. 1) [18], and their spectral difference was almost completely rooted in the D ring. Two aromatic singlets at *δ*_H_ 6.81 (s, H-7), 6.26 (s, H-10) and a methylenedioxy signal at *δ*_H_ 5.90, 5.89 (each 1H, d, *J* = 1.0 Hz) were newly detected, while the prenyl and methoxy signals disappeared, which suggested that the methylenedioxy group should be connected to C-8 and C-9. The inference was confirmed by the HMBC correlations from the proton at *δ*_H_ 6.81 (s, H-7) to the carbons at *δ*_c_ 78.7 (s, C-6a), 144.2 (s, C-8), and 151.6 (s, C-9), and from the methylenedioxy protons to the carbons at *δ*_c_ 144.2 (s, C-8), and 151.6 (s, C-9). Regrettably, it was inconclusive to establish relative configurations at C-6a, C-11a and C-11b by ROESY analysis, since the pivotal hydroxy signals were undetectable in CD_3_OD. As we know, hydroxy proton signals were observable and often appeared as sharp peaks in DMSO-*d*_6_, and their HMBC and ROESY correlations often played an important role in structure elucidation, especially the determination of relative configuration [19]. The clear ROESY correlation (DMSO-*d*_6_, Fig. 2) of 6a-OH ↔ H-11a revealed a *cis* fusion of the B/C ring junction, while the correlations of 11b-OH ↔ H-11a and H-6*α* indicated *α*-orientation of the hydroxy group at C-11b. Accordingly, the structure of **1** was established and named as pterocarpadiol A.

**Table 1:** ^1^H NMR spectroscopic data for pterocarpadiols A–D (**1**–**4**) in CD_3_OD (*δ*_H_ 3.30 ppm)

No.	**1**	**2**	**3**	**4**
1	6.80 (d, 10.0)	6.83 (d, 10.0)	2.64 (td, 13.9, 4.5, H*β*) 2.01 (ddd, 13.9, 4.5, 2.5, H*α*)	2.68 (td, 13.9, 4.0, H*β*) 2.03 (ddd, 13.9, 5.0, 2.0, H*α*)
2	6.09 (dd, 10.0, 1.8)	6.10 (dd, 10.0, 1.8)	2.76 (ddd, 16.5, 13.9, 4.5, H*α*) 2.31 (ddd, 16.5, 4.5, 2.5, H*β*)	2.77 (ddd, 16.2, 13.9, 5.0, H*α*) 2.31 (ddd, 16.2, 4.0, 2.0, H*β*)
4	5.41 (d, 1.8)	5.39 (d, 1.8)	5.35 (s)	5.34 (s)
6	4.99 (d, 10.5, H*α*) 4.37 (d, 10.5, H*β*)	5.02 (d, 10.6, H*α*) 4.39 (d, 10.6, H*β*)	4.65 (d, 10.0, H*α*) 4.30 (d, 10.0, H*β*)	4.68 (d, 10.0, H*α*) 4.32 (d, 10.0, H*β*)
7	6.81 (s)	7.25 (d, 8.5)	6.81 (s)	7.25 (d, 8.0)
8		6.55 (dd, 8.5, 2.4)		6.56 (dd, 8.0, 2.0)
10	6.26 (s)	6.29 (d, 2.4)	6.34 (s)	6.36 (d, 2.0)
11a	4.73 (s)	4.75 (s)	4.48 (s)	4.51 (s)
OCH_2_O	5.90 (d, 1.0) 5.89 (d, 1.0)		5.91 (s) 5.89 (s)	
OCH_3_		3.72 (s)		3.74 (s)

Compound **2**, white amorphous powder, had a molecular formula of C_16_H_14_O_6_ based on the positive-ion HRESIMS at *m/z* 325.0674 [M+Na]^+^ (calcd for C_16_H_14_O_6_Na, 325.0683). The NMR spectroscopic data (Tables 1, 3) were similar to those of pterocarpadiol A (**1**), and the major difference was that its NMR spectra newly displayed a methoxy group (*δ*_H_ 3.72; *δ*_c_ 56.0) instead of the methylenedioxy. And then three aromatic protons at *δ*_H_ 7.25 (d, *J* = 8.5 Hz, H-7), 6.55 (dd, *J* = 8.5, 2.4 Hz, H-8), and 6.29 (d, *J* = 2.4 Hz, H-10) were assignable to an ABX-type aromatic D-ring. The methoxy group was linked to C-9 on the basis of the HMBC correlations from the proton at *δ*_H_ 7.25 (d, *J* = 8.5 Hz, H-7) to the carbons at *δ*_c_ 78.0 (s, C-6a), 164.2 (s, C-9), and 162.9 (s, C-10a), and from the methoxy protons at *δ*_H_ 3.72 (s) to the carbon at *δ*_c_ 164.2 (s, C-9). Therefore, the structure of **2** was established and named as pterocarpadiol B.

Compound **3**, white amorphous powder, possessed a molecular formula of C_16_H_14_O_7_ according to its positive-ion HRESIMS at *m/z* 341.0620 [M+Na]^+^ (calcd for C_16_H_14_O_7_Na, 341.0632), which was 2 m.u. higher than that of **1**. Signals of an oxymethylene at *δ*_H_ 4.49, 4.28 (each 1H, d, *J* = 10.0 Hz, H-6), an oxymethine at *δ*_H_ 4.44 (s, H-11a), an olefinic proton at *δ*_H_ 5.22 (s, H-4), two aromatic singlets at 6.93 (s, H-7), 6.49 (s, H-10) and a methylenedioxy at *δ*_H_ 5.96, 5.94 (each 1H, s) were observed in the ^1^H NMR spectrum (Table 2). By comparison of the NMR spectra (Tables 2, 3) of **3** and **1**, two *ortho*-coupled doublets and the corresponding olefinic carbons were absent whereas two *sp*^3^ carbons at *δ*_c_ 31.2 (t) and *δ*_c_ 31.8 (t) were newly detected, which hinted that **3** should be 1,2-dihydropterocarpadiol A. The inference was confirmed by the HMBC correlations from the proton at *δ*_H_ 2.43 (td, 14.1, 4.5, H-1*β*) to the carbon at *δ*_c_ 89.9 (d, C-11a) and from the proton at *δ*_H_ 2.16 (ddd, 16.0, 4.5, 2.8, H-2*β*) to the carbon at *δ*_c_ 107.5 (d, C-4). The clear ROESY correlations (DMSO-*d*_6_) of 6a-OH/11b-OH ↔ H-11a indicated that **3** possessed the same stereochemistry as **1**. Thus, the structure of **3** was established and named as pterocarpadiol C.

**Table 2:** ^1^H NMR spectroscopic data for pterocarpadiol A (**1**) and pterocarpadiol C (**3**) in DMSO-*d*_6_ (*δ*_H_ 2.49 ppm)

No.	**1**	**3**
1	6.79 (d, 10.0)	2.43 (td, 14.1, 4.5, H*β*) 1.93 (ddd, 14.1, 4.9, 2.8, H*α*)
2	6.02 (dd, 10.0, 1.3)	2.65 (ddd, 16.0, 14.1, 4.9, H*α*) 2.16 (ddd, 16.0, 4.5, 2.8, H*β*)
4	5.32 (d, 1.3)	5.22 (s)
6	4.79 (d, 10.1, H*α*) 4.36 (d, 10.1, H*β*)	4.49 (d, 10.0, H*α*) 4.28 (d, 10.0, H*β*)
7	6.93 (s)	6.93 (s)
10	6.44 (s)	6.49 (s)
11a	4.68 (s)	4.44 (s)
OCH_2_O	5.94 (s) 5.93 (s)	5.96 (s) 5.94 (s)
OH-6a	6.40 (s)	6.31 (s)
OH-11b	6.77 (s)	6.27 (s)

**Table 3:** ^13^C NMR spectroscopic data for pterocarpadiols A–D (**1**–**4**)

No.	**1** ^a^	**2** ^a^	**3** ^a^	**4** ^a^	**1** ^b^	**3** ^b^
1	146.5 d	146.5 d	32.7 t	32.8 t	144.9 d	31.2 t
2	128.9 d	128.9 d	32.8 t	32.8 t	127.8 d	31.8 t
3	190.2 s	190.2 s	202.1 s	202.1 s	187.0 s	197.7 s
4	106.9 d	106.9 d	108.9 d	108.9 d	105.8 d	107.5 d
4a	172.9 s	172.9 s	174.2 s	174.1 s	169.8 s	170.9 s
6	70.2 t	70.4 t	70.5 t	70.8 t	68.7 t	68.9 t
6a	78.7 s	78.0 s	78.8 s	78.1 s	76.9 s	76.9 s
6b	119.9 s	120.9 s	121.0 s	122.1 s	119.7 s	120.6 s
7	104.1 d	125.6 d	104.1 d	125.5 d	103.9 d	103.8 d
8	144.2 s	109.5 d	144.2 s	109.4 d	142.1 s	142.0 s
9	151.6 s	164.2 s	151.4 s	164.1 s	149.4 s	149.1 s
10	93.6 d	96.4 d	93.6 d	96.6 d	92.7 d	92.6 d
10a	156.5 s	162.9 s	155.8 s	162.2 s	154.5 s	153.7 s
11a	91.4 d	91.4 d	91.8 d	91.8 d	89.6 d	89.9 d
11b	68.8 s	68.8 s	69.7 s	69.7 s	67.1 s	67.9 s
OCH_2_O	103.0 t		102.9 t		101.6 t	101.4 t
OCH_3_		56.0 q		56.0 q		

^a^Measured in CD_3_OD (*δ*_c_ 49.0 ppm)

^b^Measured in DMSO-*d*_6_ (*δ*_c_ 39.5 ppm)

Compound **4**, white amorphous powder, had a molecular formula of C_16_H_16_O_6_ determined by the positive-ion HRESIMS at *m/z* 327.0819 [M+Na]^+^ (calcd for C_16_H_16_O_6_Na, 327.0839). The NMR spectroscopic data (Tables 1, 3) were similar to those of pterocarpadiol C (**3**), and the obvious difference was that a methoxy signal (*δ*_H_ 3.74; *δ*_c_ 56.0) replaced the methylenedioxy in **3**. According to the HMBC correlations from the proton at *δ*_H_ 7.25 (d, *J* = 8.0 Hz, H-7) to the carbons at *δ*_c_ 78.1 (s, C-6a), 164.1 (s, C-9), and 162.2 (s, C-10a), and from the methoxy protons at *δ*_H_ 3.74 (s) to the carbon at *δ*_c_ 164.1 (s, C-9), the methoxy group was positioned at C-9 as with the previous structure. Thus, the structure of **4** was established and named as pterocarpadiol D.

Pterocarpans isolated in our current research such as pisatin (**7**), variabilin (**8**), and maackiain (**11**) exhibited without exception negative optical rotation values (−286°, −304°, and −260°, respectively), and their absolute configurations had been established as 6a*S*,11a*S* (**7**), 6a*S*,11a*S* (**8**), and 6a*R*,11a*R* (**11**) [20]. As their related co-constituents, pterocarpadiols A–D (**1**–**4**) also gave large negative optical rotation values (−484.0°, −397.0°, −507.0° and −476.0°, respectively), thereupon we assumed that the absolute configurations of **1**–**4** could be assigned as 6a*S*,11a*R*,11b*S* depicted in Fig. 1. Nonetheless, this issue deserved further studies in the future. Until now, only two 6a,11b-dihydroxypterocarpans: hydroxytuberosone [21] and hydroxycristacarpone [18], were reported and only from the family Leguminosae, therefore pterocarpadiols A–D as chemical markers in *Derris* species may be helpful in chemotaxonomical classification.

## Experimental Section

### General Experimental Procedures

Optical rotations were measured on a Jasco P-1020 automatic digital polarimeter. UV data were obtained from HPLC online analysis. NMR spectra were carried out on a Bruker AV-400, Bruker DRX-500 or Bruker AV-800 spectrometer with deuterated solvent signals used as internal standards. ESI and HRESIMS were performed with a Shimadzu LC-IT-TOF mass spectrometer equipped with an ESI interface (Shimadzu, Kyoto, Japan). Silica gel 200–300 mesh (Qingdao Marine Chemical Inc., Qingdao, China), Chromatorex C-18 (40–75 μm, Fuji Silysia Chemical Ltd., Japan) and Sephadex LH-20 (Amersham Biosciences, Uppsala, Sweden) were used for normal pressure column chromatography (CC). Fractions were monitored and analyzed by TLC, in combination with Agilent 1200 series HPLC system equipped by Extend-C18 column (5 *μ*m, 4.6 × 150 mm).

### Plant Material

The twigs and leaves of *D.robusta* were collected from the Pu’er region of Yunnan Province, China, in May 2011, and identified by Mr. Yu Chen of Kunming Institute of Botany. A voucher specimen (BBP0350021DR) was deposited at BioBioPha Co., Ltd.

### Extraction and Isolation

The air-dried and powdered twigs and leaves (12.0 kg) of *D.robusta* were extracted with 95 % EtOH at room temperature, and the solvent was removed under reduced pressure to give crude extract (ca. 870 g), which was fractionated by silica gel CC successively eluted with petroleum ether (PE)/acetone gradient and then MeOH to yield nine fractions A–I. Fraction C (PE/acetone, 6:1) was subjected to silica gel CC (CHCl_3_/MeOH, 100:0 → 100:2) and Sephadex LH-20 (CHCl_3_/MeOH, 1:1; or MeOH) to give **5** (51 mg), **6** (185 mg), **7** (138 mg), **8** (262 mg), **11** (665 mg), and **12** (608 mg), and the remainder was separated by RP-18 (40 % MeOH/H_2_O) and Sephadex LH-20 (MeOH) to yielded **9** (661 mg) and **10** (349 mg). The fraction E (PE/acetone, 4:1) was repeatedly applied to silica gel CC (CHCl_3_/MeOH, 30:1 → 15:1) and Sephadex LH-20 (MeOH) to yiele **13** (23 mg) and **14** (114 mg). The fraction F (PE/acetone, 3:1) was repeatedly separated on silica gel CC (CHCl_3_/MeOH, 10:1), Sephadex LH-20 (CHCl_3_/MeOH, 1:1) and RP-18 (40 % MeOH/H_2_O) to yield **1** (233 mg) and **2** (33 mg), and the remainder was further isolated on silica gel (CHCl_3_/MeOH, 10:1), Sephadex LH-20 (MeOH) and RP-18 (45 % MeOH/H_2_O) to yield **3** (47 mg) and **4** (28 mg). The retention times (*t*_R_) of **1**–**4** on an analytical HPLC Extend-C18 column (20 % → 100 % MeOH in H_2_O over 8.0 min followed by 100 % MeOH to 13.0 min, 1.0 ml/min, 25 °C) were 6.03, 6.16, 6.50, and 6.60 min, respectively.

### Pterocarpadiol A (**1**)

White amorphous powder; UV (MeOH) *λ*_max_: 235, 306 nm; $\left[ \alpha \right]_{\text{D}}^{23}$ −484.0 (*c* 0.5, MeOH); ^1^H NMR data: see Tables 1 and 2; ^13^C NMR data: see Table 3; ESIMS (pos.): *m/z* 339 [M+Na]^+^; HRESIMS (pos.): *m/z* 339.0462 [M+Na]^+^ (calcd for C_16_H_12_O_7_Na, 339.0475).

### Pterocarpadiol B (**2**)

White amorphous powder; UV (MeOH) *λ*_max_: 230, 285, 305 nm; $\left[ \alpha \right]_{\text{D}}^{22}$ −397.0 (*c* 0.2, MeOH); ^1^H NMR data: see Table 1; ^13^C NMR data: see Table 3; ESIMS (pos.): *m/z* 325 [M+Na]^+^; HRESIMS (pos.): *m/z* 325.0674 [M+Na]^+^ (calcd for C_16_H_14_O_6_Na, 325.0683).

### Pterocarpadiol C (**3**)

White amorphous powder, UV (MeOH) *λ*_max_: 260, 308 nm; $\left[ \alpha \right]_{\text{D}}^{23}$ −507.0 (*c* 0.2, MeOH); ^1^H NMR data: see Tables 1 and 2; ^13^C NMR data: see Table 3; ESIMS (pos.): *m/z* 341 [M+Na]^+^; HRESIMS (pos.): *m/z* 341.0620 [M+Na]^+^ (calcd for C_16_H_14_O_7_Na, 341.0632).

### Pterocarpadiol D (**4**)

White amorphous powder, UV (MeOH) *λ*_max_: 232, 261 nm; $\left[ \alpha \right]_{\text{D}}^{23}$ −476.0 (*c* 0.2, MeOH); ^1^H NMR data: see Table 1; ^13^C NMR data: see Table 3; ESIMS (pos.): *m/z* 327 [M+Na]^+^; HRESIMS (pos.): *m/z* 327.0819 [M+Na]^+^ (calcd for C_16_H_16_O_6_Na, 327.0839).

## Electronic supplementary material

Supplementary material 1 (DOCX 1223 kb)
