# Supplementary material for: Pterocarpadiols A–D, Rare 6a,11b-Dihydroxypterocarpans from Derris robusta
Source: Nat Prod Bioprospect. 2015 Nov 7;5(6):287–91. doi: 10.1007/s13659-015-0078-y (PMC4681708; doi:10.1007/s13659-015-0078-y)

Regular Article *Nat. Prod. Bioprospect.*

**Pterocarpadiols A–D,** **rare 6a,11b-dihydroxypterocarpans from** ***Derris robusta***

Xiang-Mei Li • Mei-Fen Mao • Fu-Cai Ren • Xian-Jun Jiang • Ping Hai • Fei Wang*

BioBioPha Co., Ltd.; Kunming 650201, China

* To whom correspondence should be addressed.

Email: [f.wang@mail.biobiopha.com](mailto:f.wang@mail.biobiopha.com)

**Fig. 1** Structures of pterocarpadiols A–D (**1**–**4**)

**Content list:**

**S1.** ^1^H NMR spectrum (500 MHz, CD_3_OD) of pterocarpadiol A (**1**).

**S2.** ^13^C NMR spectrum (100 MHz, CD_3_OD) of pterocarpadiol A (**1**).

**S3.** HMBC spectrum (500 MHz, CD_3_OD) of pterocarpadiol A (**1**).

**S4.** ^1^H NMR spectrum (800 MHz, DMSO-*d*_6_) of pterocarpadiol A (**1**).

**S5.** ^13^C NMR spectrum (200 MHz, DMSO-*d*_6_) of pterocarpadiol A (**1**).

**S6.** HMBC spectrum (800 MHz, DMSO-*d*_6_) of pterocarpadiol A (**1**).

**S7.** ROESY spectrum (800 MHz, DMSO-*d*_6_) of pterocarpadiol A (**1**).

**S8.** ^1^H NMR spectrum (500 MHz, CD_3_OD) of pterocarpadiol B (**2**).

**S9.** ^13^C NMR spectrum (100 MHz, CD_3_OD) of pterocarpadiol B (**2**).

**S10.** HMBC spectrum (500 MHz, CD_3_OD) of pterocarpadiol B (**2**).

**S11.** ^1^H NMR spectrum (500 MHz, CD_3_OD) of pterocarpadiol C (**3**).

**S12.** ^13^C NMR spectrum (125 MHz, CD_3_OD) of pterocarpadiol C (**3**).

**S13.** ^1^H NMR spectrum (500 MHz, DMSO-*d*_6_) of pterocarpadiol C (**3**).

**S14.** ^13^C NMR spectrum (100 MHz, DMSO-*d*_6_) of pterocarpadiol C (**3**).

**S15.** HMBC spectrum (500 MHz, DMSO-*d*_6_) of pterocarpadiol C (**3**).

**S16.** ROESY spectrum (500 MHz, DMSO-*d*_6_) of pterocarpadiol C (**3**).

**S17.** ^1^H NMR spectrum (500 MHz, CD_3_OD) of pterocarpadiol D (**4**).

**S18.** ^13^C NMR spectrum (125 MHz, CD_3_OD) of pterocarpadiol D (**4**).

**S19.** HMBC spectrum (500 MHz, CD_3_OD) of pterocarpadiol D (**4**).

**S1.** ^1^H NMR spectrum (500 MHz, CD_3_OD) of pterocarpadiol A (**1**).


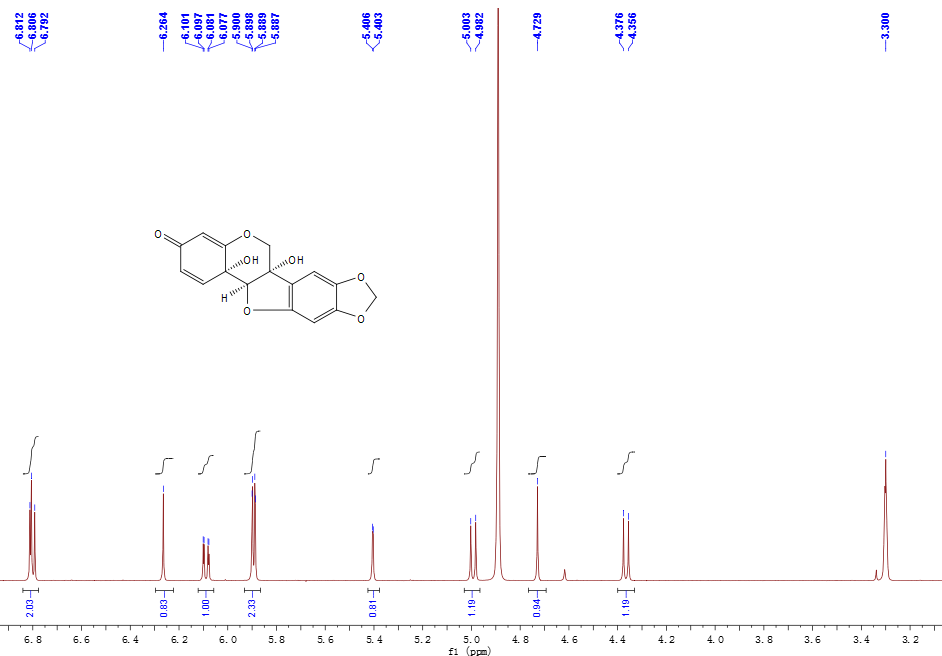


**S2.** ^13^C NMR spectrum (100 MHz, CD_3_OD) of pterocarpadiol A (**1**).


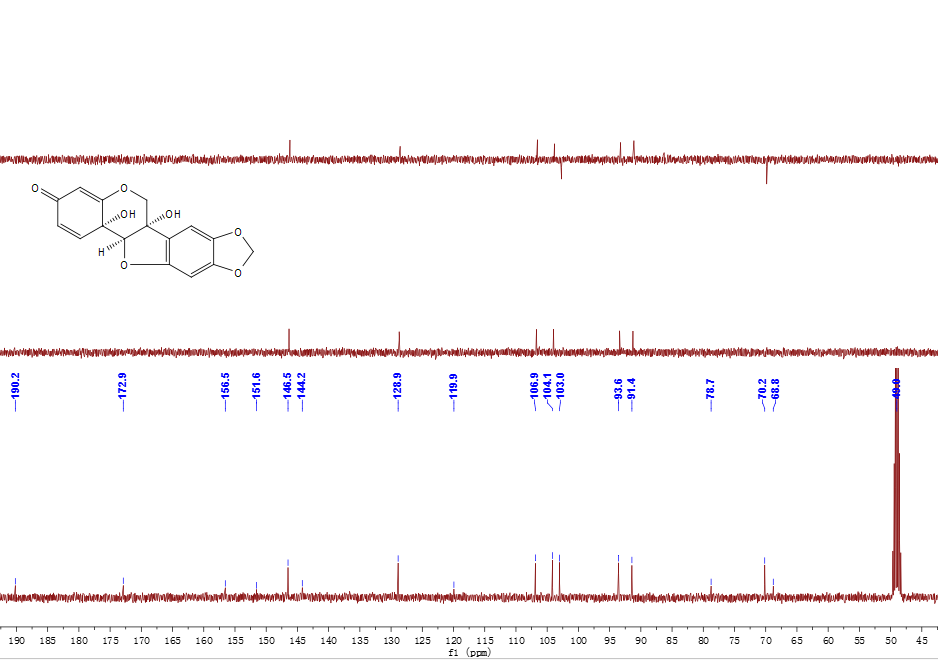


**S3.** HMBC spectrum (500 MHz, CD_3_OD) of pterocarpadiol A (**1**).


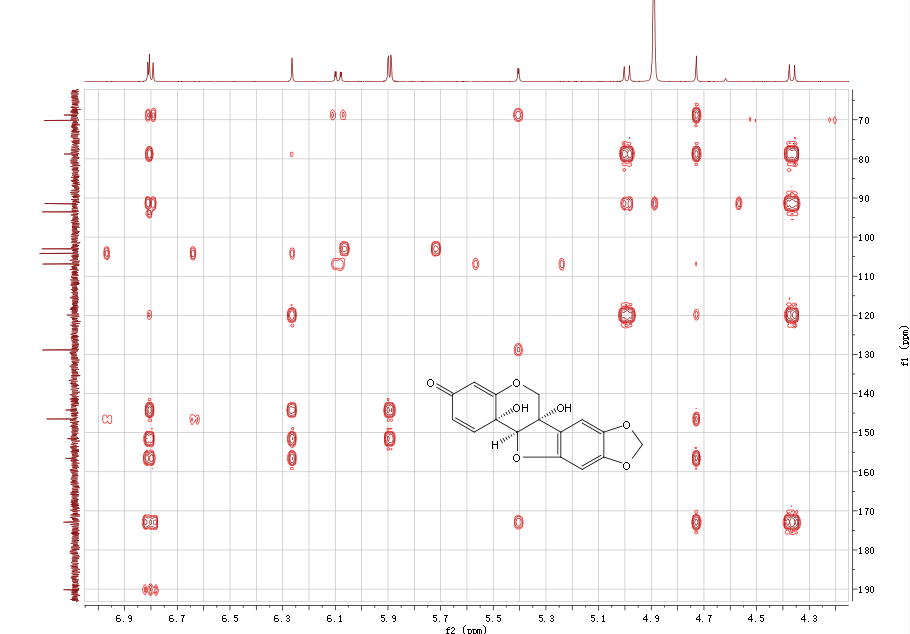


**S4.** ^1^H NMR spectrum (800 MHz, DMSO-*d*_6_) of pterocarpadiol A (**1**).


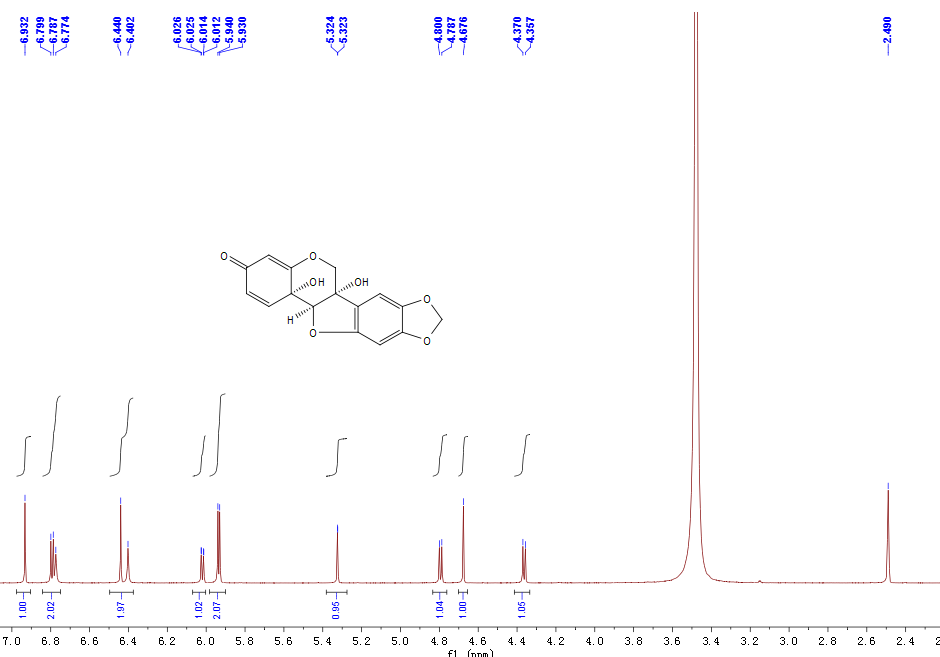


**S5.** ^13^C NMR spectrum (200 MHz, DMSO-*d*_6_) of pterocarpadiol A (**1**).


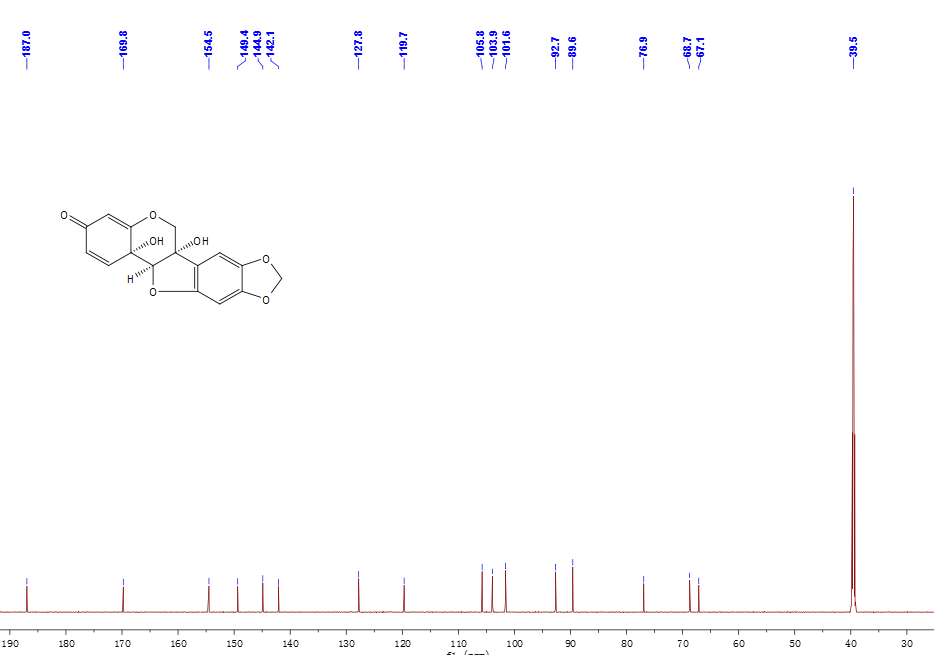


**S6.** HMBC spectrum (800 MHz, DMSO-*d*_6_) of pterocarpadiol A (**1**).


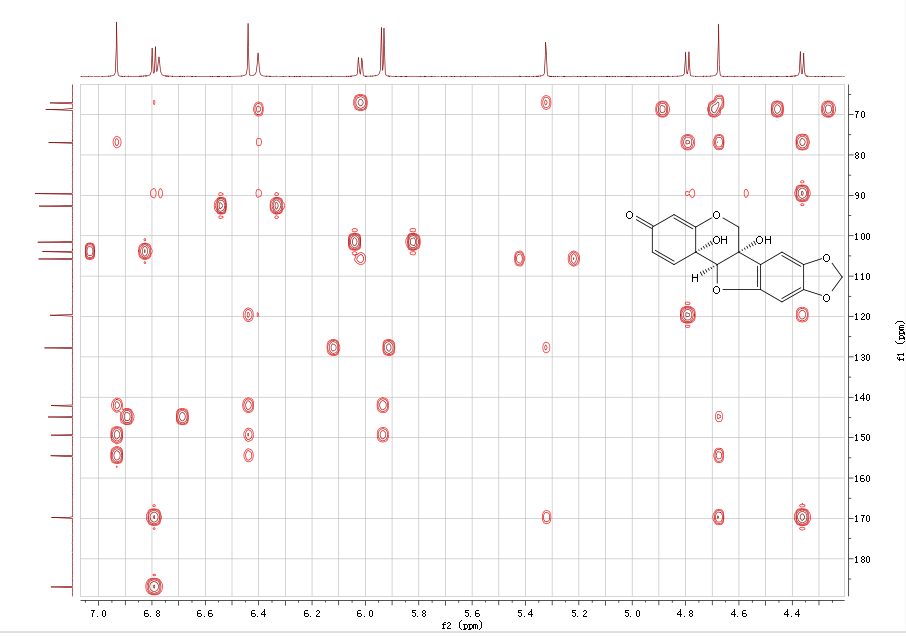


**S7.**  ROESY spectrum (800 MHz, DMSO-*d*_6_) of pterocarpadiol A (**1**).


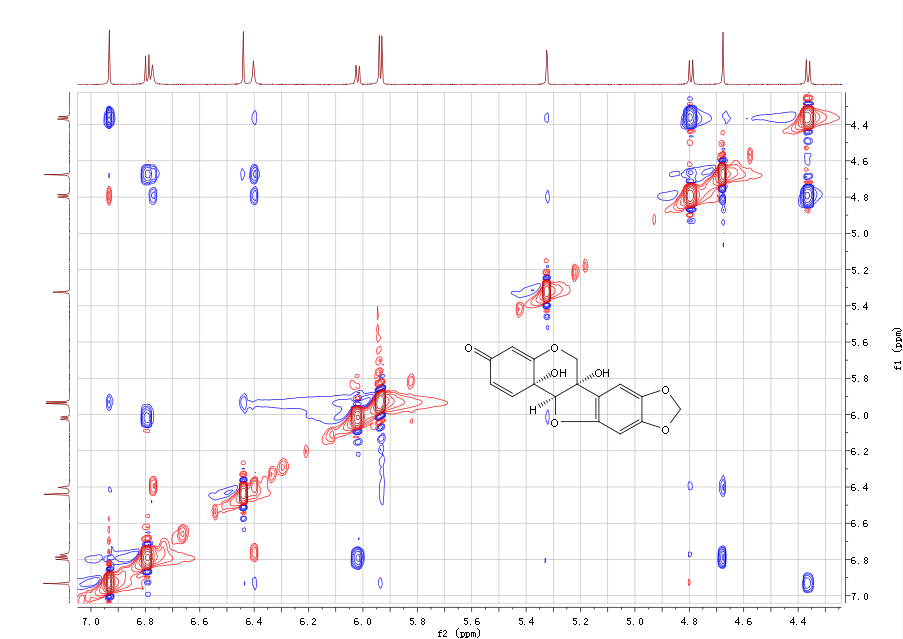


**S8.** ^1^H NMR spectrum (500 MHz, CD_3_OD) of pterocarpadiol B (**2**).


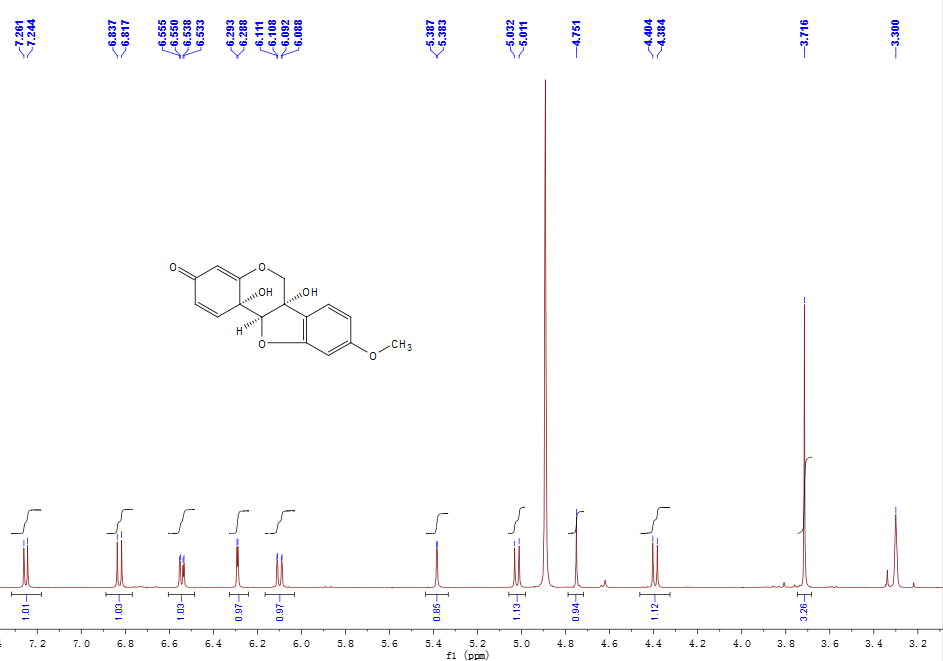


**S9.** ^13^C NMR spectrum (100 MHz, CD_3_OD) of pterocarpadiol B (**2**).


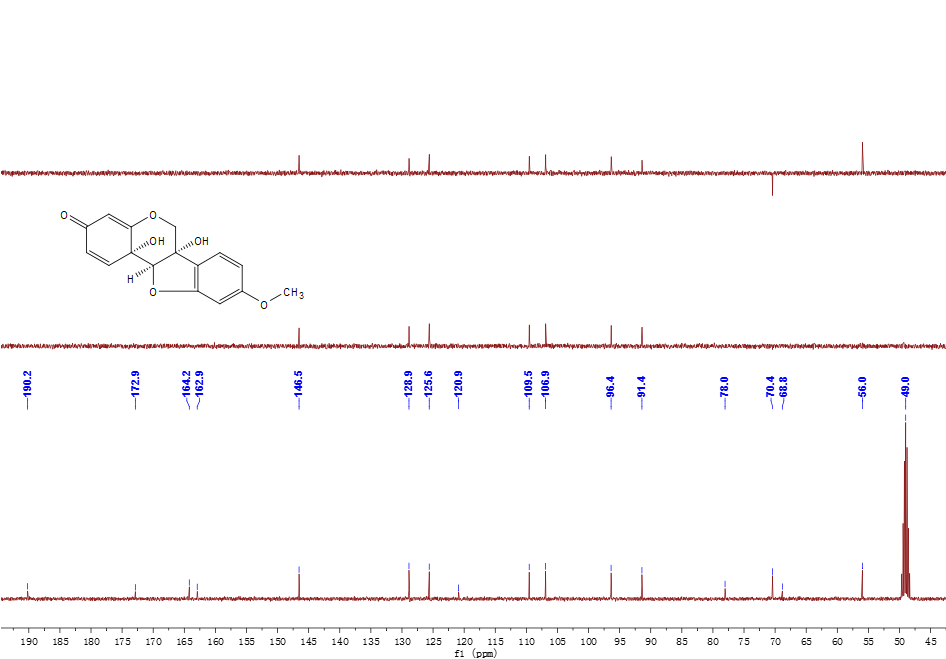


**S10.** HMBC spectrum (500 MHz, CD_3_OD) of pterocarpadiol B (**2**).


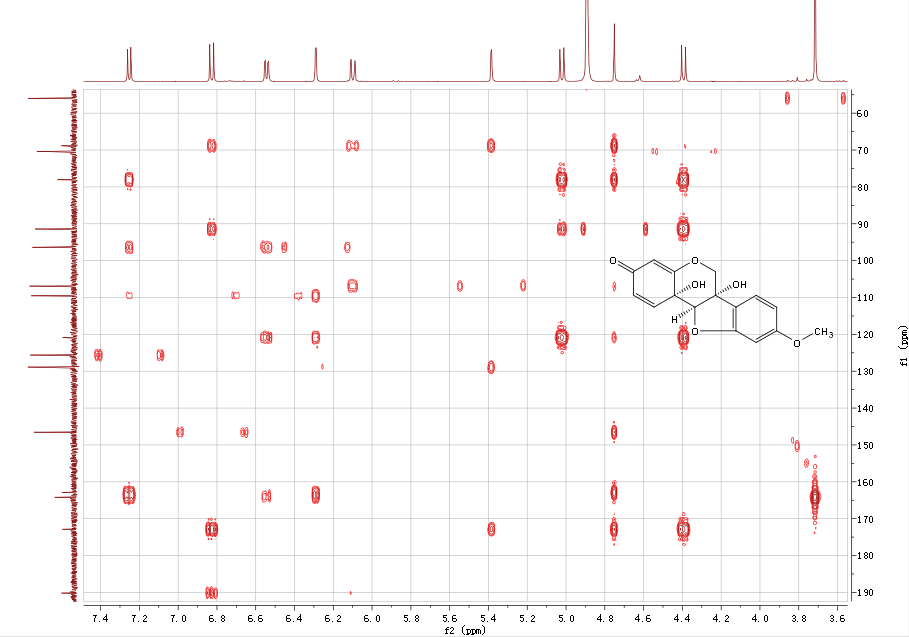


**S11.** ^1^H NMR spectrum (500 MHz, CD_3_OD) of pterocarpadiol C (**3**).


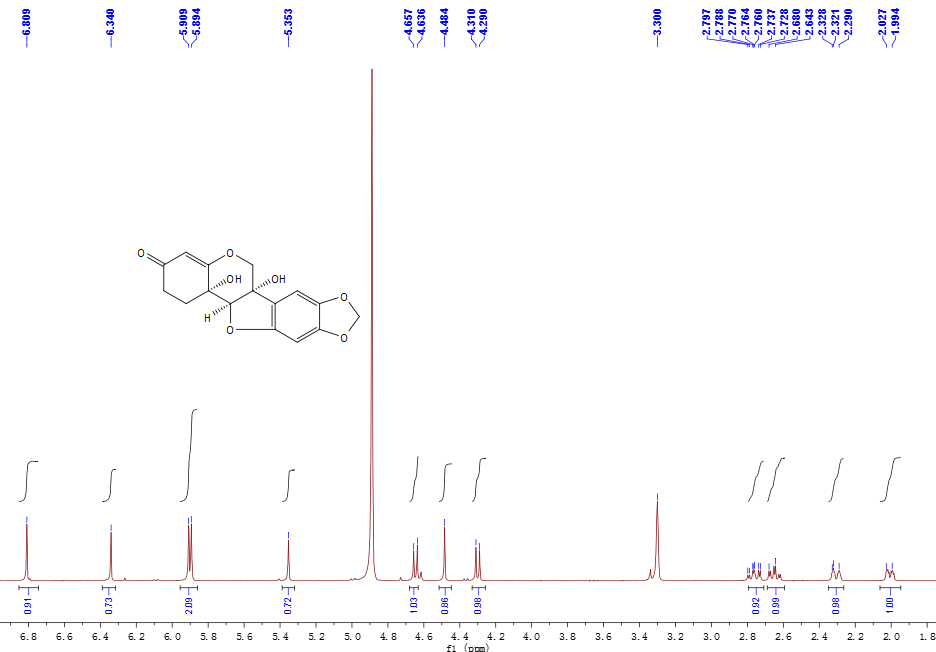


**S12.** ^13^C NMR spectrum (125 MHz, CD_3_OD) of pterocarpadiol C (**3**).


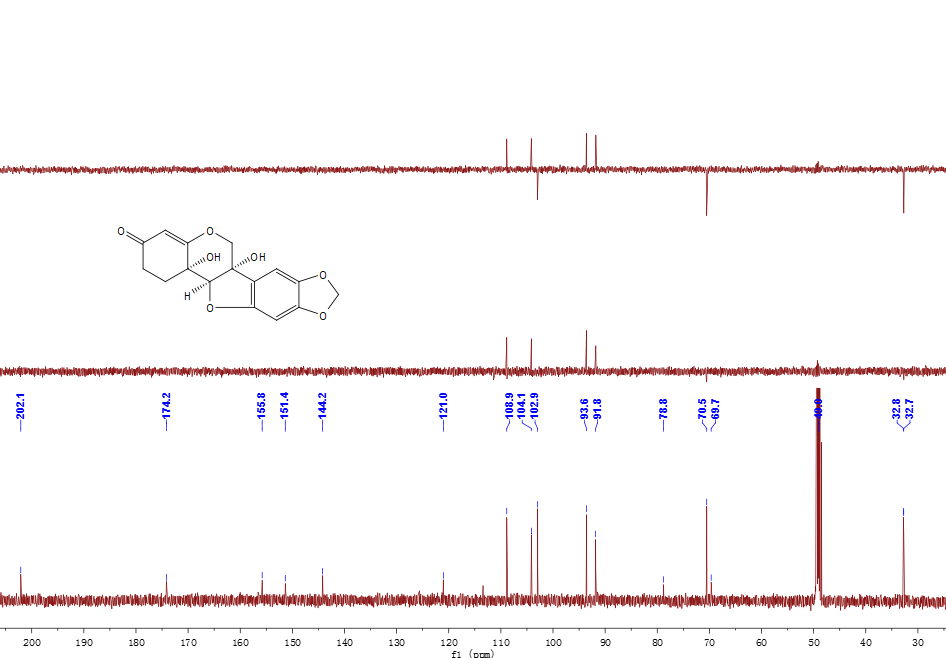


**S13.** ^1^H NMR spectrum (500 MHz, DMSO-*d*_6_) of pterocarpadiol C (**3**).


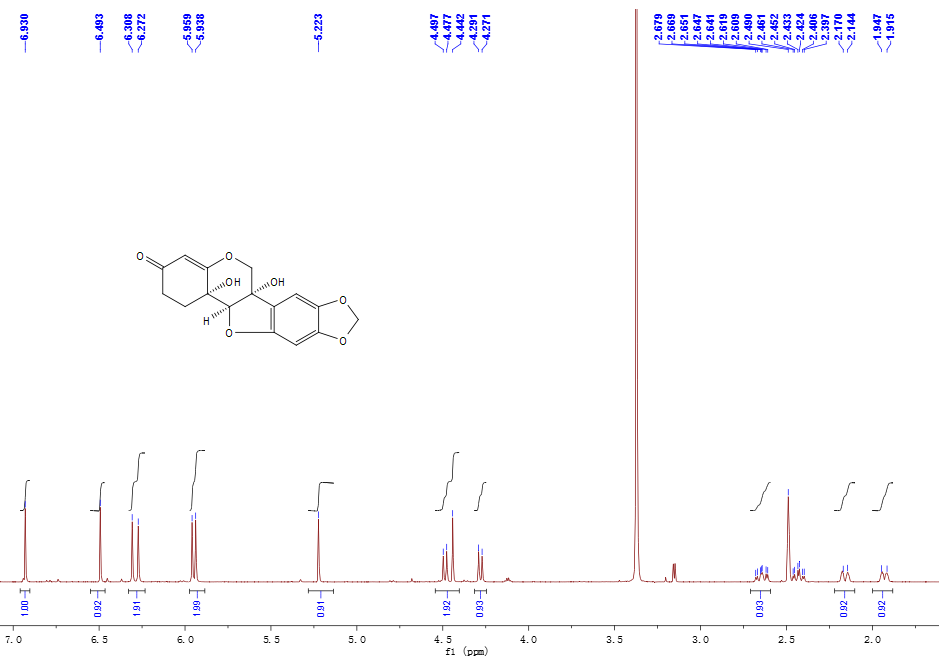


**S14.** ^13^C NMR spectrum (100 MHz, DMSO-*d*_6_) of pterocarpadiol C (**3**).


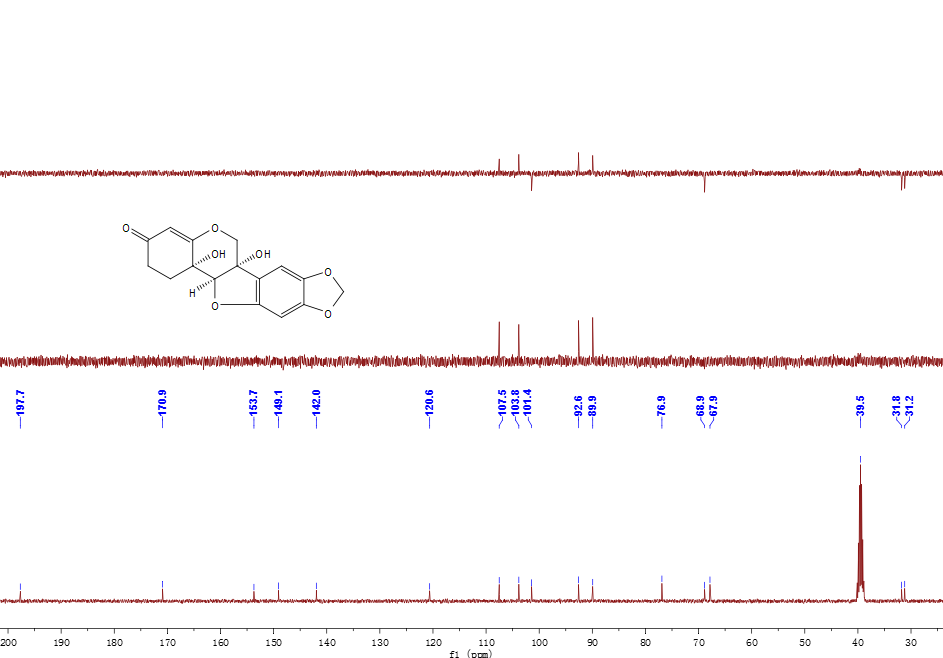


**S15.** HMBC spectrum (500 MHz, DMSO-*d*_6_) of pterocarpadiol C (**3**).


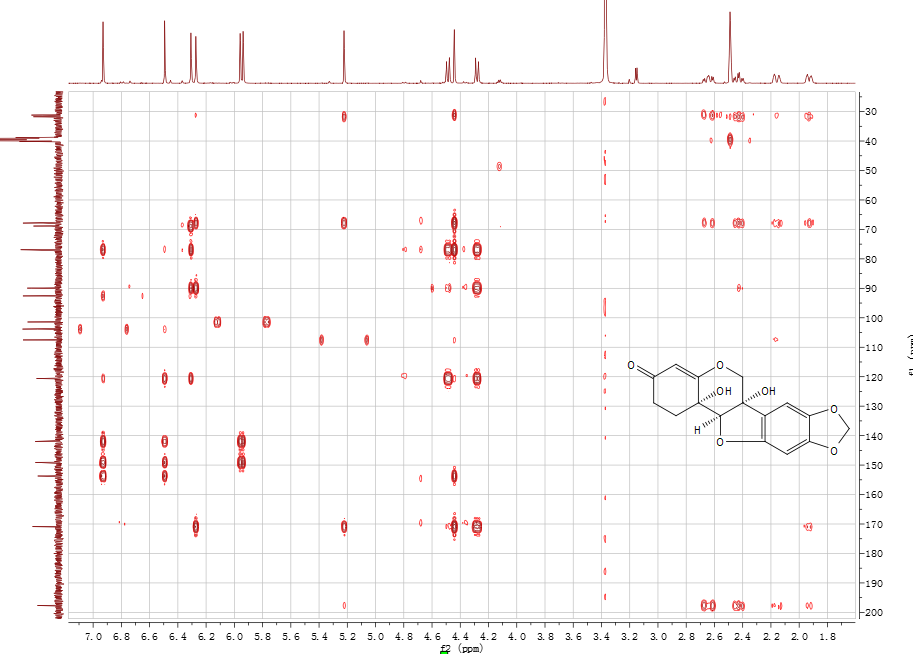


**S16.** ROESY spectrum (500 MHz, DMSO-*d*_6_) of pterocarpadiol C (**3**).


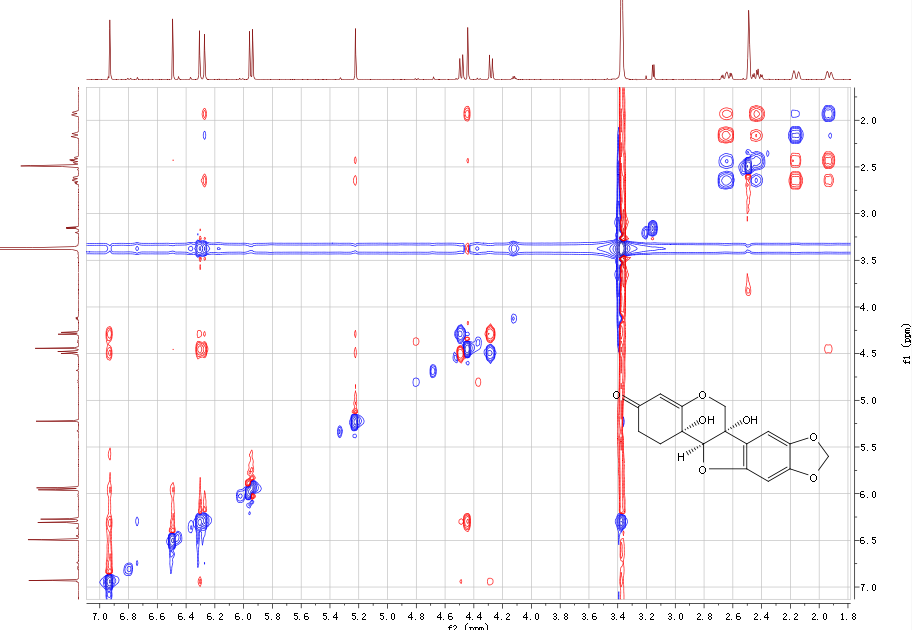


**S17.** ^1^H NMR spectrum (500 MHz, CD_3_OD) of pterocarpadiol D (**4**).


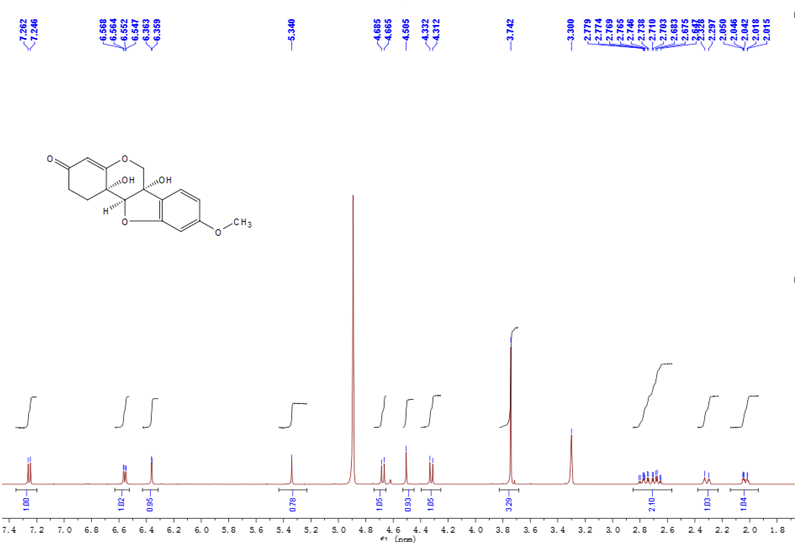


**S18.** ^13^C NMR spectrum (125 MHz, CD_3_OD) of pterocarpadiol D (**4**).


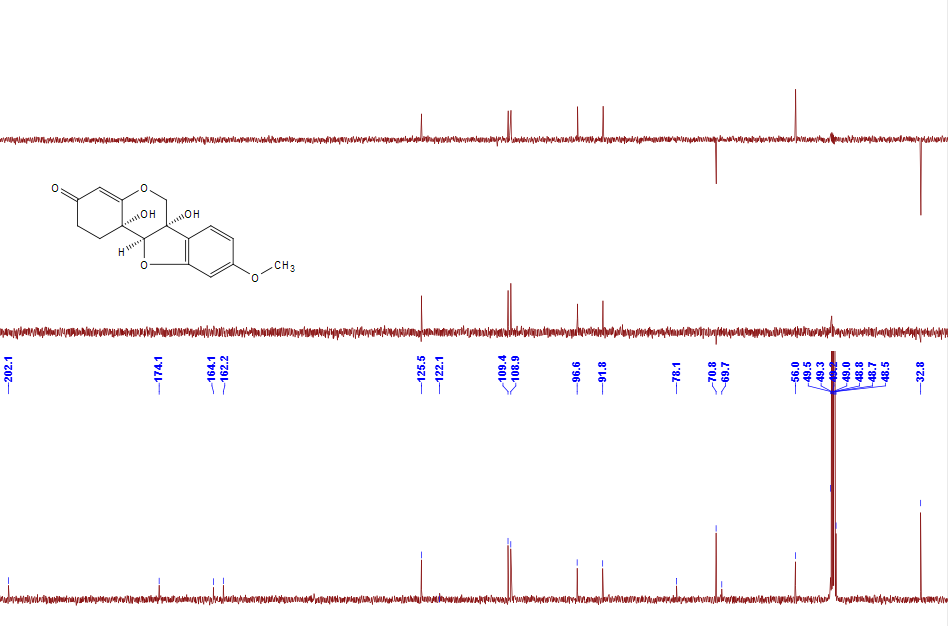


**S19.** HMBC spectrum (500 MHz, CD_3_OD) of pterocarpadiol D (**4**).


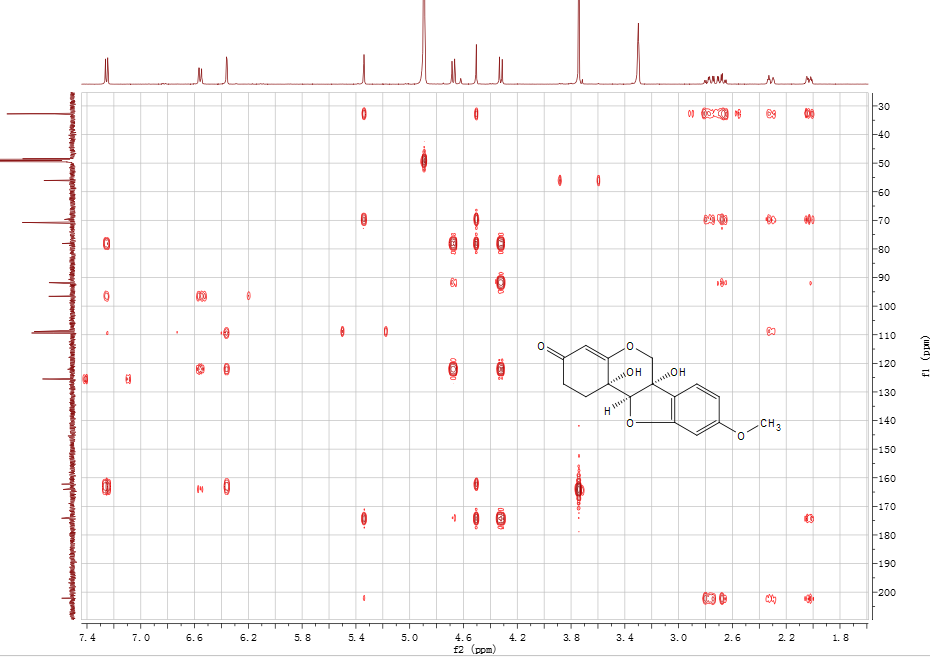

Supplement: Supplementary file 1 — Supplementary material 1 (DOCX 1223 kb) [file 13659_2015_78_MOESM1_ESM.docx]
